# Meta-analysis comparing maintenance strategies with continuous therapy and complete chemotherapy-free interval strategies in the treatment of metastatic colorectal cancer

**DOI:** 10.18632/oncotarget.8644

**Published:** 2016-04-08

**Authors:** Lei Zhao, Jing Wang, Huihui Li, Juanjuan Che, Bangwei Cao

**Affiliations:** ^1^ Department of Oncology, Beijing Friendship Hospital, Capital Medical University, Beijing, China

**Keywords:** randomized controlled trials, grade 3 to 4 toxicities, pooled analysis, systematic review, optimal maintenance strategies

## Abstract

There is as yet no consensus as to the best choice among the three treatment options (maintenance, complete chemotherapy-free intervals [CFIs], and continuous) for metastatic colorectal cancer (CRC). We performed a meta-analysis of six trials (*N* = 2, 454 patients) to compare the safety and efficacy of those three treatment strategies. Maintenance appeared to offer an advantage over CFI with respect to progression-free survival (PFS) (hazard ratio [HR]: 0.53, 95% confidence interval [CI], 0.40–0.69). PFS and overall survival (OS) were comparable between the maintenance and continuous strategies (HR: 1.18, 95% CI, 0.96–1.46; HR: 1.05, 95% CI, 0.98–1.27, respectively), as was OS between the maintenance and CFI strategies (HR: 0.84; 95% CI, 0.70–1.00). The incidence of grade 3/4 toxicity, including neutropenia, neuropathy, hand-foot syndrome and fatigue, was lower with maintenance than with continuous therapy. A maintenance regimen utilizing bevacizumab-based doublets appeared to confer a slight advantage over bevacizumab monotherapy with respect to PFS (*P* = 0.011). Maintenance appeared to reduce cumulative grade 3/4 toxicity as compared to the continuous strategy, while showing comparable efficacy. Bevacizumab-based doublets appeared to be of particular value in patients with metastatic CRC.

## INTRODUCTION

Colorectal cancer (CRC) is a common malignancy that accounts for approximately 1.2 million new cases and 600,000 deaths worldwide every year [1]. Approximately 40–50% of CRC patients develop metastatic disease. While surgery is the cornerstone treatment for early-stage CRC (stage I–III), chemotherapy is often the first resort in patients with metastatic disease (stage IV), when the lesions are often not fully resectable. During the past few decades, the treatment of metastatic (m)CRC has undergone significant advances, resulting in improved outcomes and prolonged survival [2]. The introduction of newer drugs such as irinotecan (CPT-11) and oxaliplatin (OHP), and biological agents such as bevacizumab (Bev) and cetuximab (Cet), has increased the response rates up to 50–60% alongside increased median progression-free survival (PFS) and overall survival (OS) (9 to 11 months and up to 30 months, respectively) [3–5].

Despite this significant progress, however, the optimal duration of treatment remains a controversial issue. For patients benefiting from standard induction chemotherapy, continuous long-term chemotherapy is inevitably associated with side effects and carries a risk of development of drug resistance. On the other hand, intermittent treatment is likely to adversely impact the chemotherapeutic efficacy and treatment outcomes. Amelioration of treatment toxicity and determination of the optimal treatment duration are thus active areas of cancer research. One of the strategies to reduce side effects is the stop-and-go strategy, which involves stopping all agents after a predefined number of cycles (3–6 months) (complete chemotherapy-free interval [CFI]) and restarting on progression. However, studies do not support CFI due to significantly reduced response rates, suggesting complete CFI may not be an optimal option for patients with mCRC [6, 7]. This issue and the status and prospects of maintenance therapies in mCRC were focuses of deliberation at the European Society for Medical Oncology, 2014. During that meeting, Professor Dirk Arnold proposed a maintenance strategy akin to that usually considered for the treatment of lung cancer.

The concept of maintenance treatment envisages a period of high-intensity chemotherapy, after which those agents that are mainly responsible for cumulative toxicity are stopped. A comparative assessment of the maintenance, complete CFI and continuous strategies may help identify the optimal chemotherapeutic regimen for sequential treatment of mCRC.

## RESULTS

**Figure 1:**
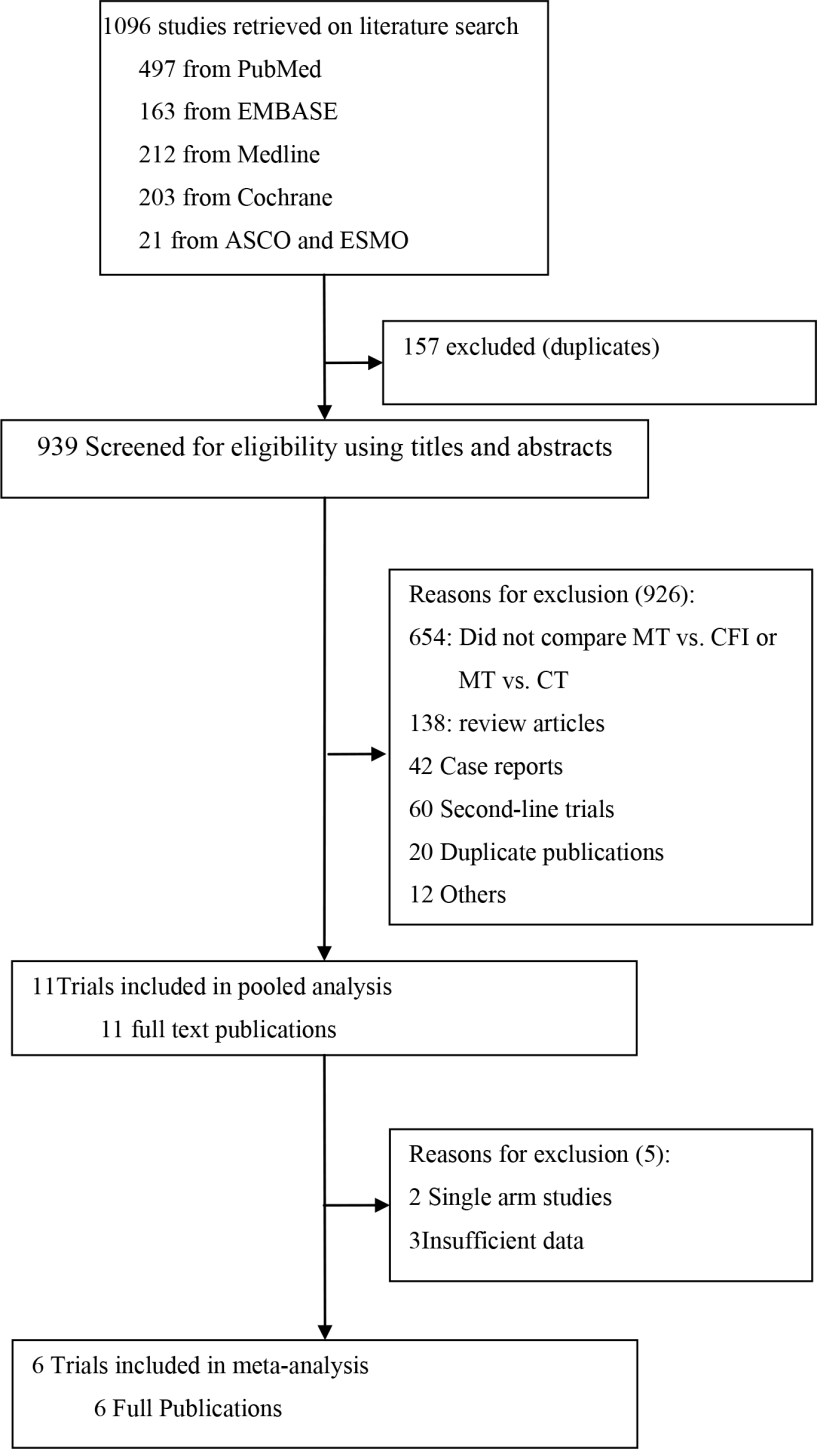
Trial selection criteria MT, maintenance therapy; CT, continuous therapy.

A total of 1,096 articles were retrieved on initial search query. Of these, 1084 studies were excluded after a review of the study titles and abstracts. After a full-text review of 11 potentially eligible articles, six studies [8–13] with a combined study population of 2454 patients with mCRC were included in the meta-analysis (Figure 1). None of the patients had a history of treatment for metastatic disease. The baseline characteristics of the six trials are summarized in Table 1. Note that the study by Hegewisch et al. [10] had three treatment arms and that armA vs. armC and armB vs. armC were treated as two separate trials. In addition to the six articles included in the meta-analysis, data from five other trials of maintenance therapies were included in the pooled analysis [14–18]. The baseline characteristics of the five trials included in the pooled analysis are summarized in Table 2. PFS was defined as the time from maintenance randomization to progression or death (not including induction therapy time). To standardize the data, PFS values from several studies [8, 9, 12, 15, 18] were adjusted from maintenance treatment to progression or death to first disease progression or death.

**Table 1:** Characteristics of relevant studies included in meta-analysis

Source	Scope	Treatment	Sample Size	Age, Median (range), y	Outcomes and raw HRs with 95% CI	Additional survival statistics (months)
**Maintenance therapy with intermittent chemotherapy**						
Chibaudel 2009 (OPTIMOX2)	Multicenter, France	**Induction:** mFOLFOX7 every 2 weeks *6cycles; **Maintenance** (sLV5FU2; restart mFOLFOX7 at PD); **CFI** (observation; restart mFOLFOX7 at PD)	98 104	67 (35–80) 67 (31–80)	DDC, 0.71 (0.51–0.99, *P* = 0.046); PFS, 0.61 (*P* = 0.0017); OS, 0.88 (*P* = 0.42)	DDC^3^: 13.1 vs. 9.2; PFS: 8.6 vs. 6.6; OS: 23.8 vs. 19.5; ORR: 59.2% vs. 59.6%
Simkens 2015 (CAIRO3)	Multicenter, Netherlands	**Induction:** (BEV+XELOX) every 3 weeks*6 cycles; **Maintenance:** BEV (7.5 mg/kg) + Cape (625 mg/m^2^ bid); restart BEV+XELOX at PD); **CFI**: observation; restart BEV+ XELOX at PD)	278 279	63 (26–81) 64 (31–81)	PFS1, 0.40 (0.36–0.52, *P* < 0.0001); PFS2, 0.63 (0.53–0.77, *P* < 0.0001); OS, 0.83 (0.68–1.01, *P* = 0.06)	PFS1^2^: 8.5 (6.5–10.3) vs. 4.1 (3.9–4.2) PFS2^1^: 11.7 (10.1–13.3) vs. 8.5 (7.4–10.4) OS: 25.9 (23.7–28.4) vs. 22.4 (20.8–24.9)
Hegewisch 2015 (AIO 0207) (Arm A VS Arm C)	Multicenter, Germany	**Induction:** (BEV+FOLFOX) every 2 weeks*12cs/(BEV+XELOX) every 3 weeks*8 cycles; **Maintenance** (BEV+5FU; restart BEV+FOLFOX/XELOX at PD); **CFI** (observation; restart BEV+FOLFOX/XELOX at PD)	158 158	64 (25–82) 66 (32–82)	PFS, 0.48 (0.37–0.61, *P* < 0.0001); TFS, 0.76 (0.59–0.99, *P* = 0.038)	PFS^2^: 6.3 (2.8–7.6) vs. 3.5 (2.9–4.1) OS: 20.2 (17.7–24.3) vs. 23.1 (19.2–27.3)
Hegewisch 2015 (AIO 0207) (Arm B vs. Arm C)	Multicenter, Germany	**Induction:** (BEV + FOLFOX) every 2 weeks*12 cycles/(BEV+XELOX) every 3 weeks*8cycles; **Maintenance** (BEV; restart BEV+FOLFOX/XELOX at PD); **CFI** (observation; restart BEV + FOLFOX/XELOX at PD)	156 158	65 (32–82) 66 (32–82)	PFS, 0.69 (0.55–0.87, *P* = 0.0018)	PFS^2^: 4.6 (4.0–5.3) vs. 3.5 (2.9–4.1) OS: 21.9 (18.7–26.9) vs. 23.1 (19.2–27.3)
**Maintenance therapy with continuous chemotherapy**						
Tournigand 2006 (OPTIMOX1)	Multicenter, France	**Induction:** FOLFOX7 every 2 weeks*6cs; **Maintenance:** (Slv5FU2 every 2 weeks*12cs; FOLFOX7 every 2 weeks*6 cycles); **Continuous:** (FOLFOX4 every 2 weeks until PD)	309 311	64 (32–80) 65 (29–80)	DDC, 0.99 (0.81–1.15, *P* = 0.89); PFS, 1.06 (0.89–1.20, *P* = 0.47); OS, 0.93 (0.72–1.11, *P* = 0.49)	DDC: 10.6 vs. 9.0; PFS: 8.7 vs. 9.0; OS: 21.2 vs. 19.3; ORR: 59.2% vs. 58.5%
DÍAZ-RUBIO 2012 (MACRO)	Multicenter, Spain	**Induction:** (BEV+ XELOX) every 3 weeks*6 cycles **Maintenance:** BEV only until PD; **Continuous:** BEV+XELOX until PD	241 239	64 (33–82) 63 (30–80)	PFS, 1.10 (0.89–1.35) OS, 1.05 (0.85–1.30)	PFS: 9.7 (8.3–10.6) vs. 10.4 (9.4–11.9); OS: 20.0 (18.0–23.3) vs. 23.2 (19.8–26.0); ORR: 49% vs. 47%
Yalcin 2013	Multicenter, Turkey	**Induction:** (BEV+XELOX) every 3 weeks*6 cycles; **Maintenance:** BEV+Cape until PD); **Continuous:** BEV+XELOX until PD	61 62	56 (34–82) 59 (25–77)	PFS, 1.67 (NR), *P* = 0.002 OS, NR, *P* = 0.100	PFS: 11.0 (9.1–12.9) vs. 8.3 (7.1–9.5); OS: 23.8 (22.0–28.8) vs. 20.2 (18.4–23.5); ORR: 66.7% vs. 59.0%

Abbreviations: BEV = Bevacizumab; Cape = capecitabine; Cet = cetuximab; CFI = chemotherapy-free interval; FLOX = 5-FU/leucovorin/oxaliplatin; FOLFOX = folinic acid(leucovorin)/5-FU/oxaliplatin; m = modified; mos = months; NR = not reported; PD = disease progression; 5FU = 5-fluorouracil; PFS = progression-free survival; OS = overall survival; DDC = duration of disease control; RR = objective response rate.

1 From randomization date to second disease progression.

2 From randomization to disease progression or death (not including induction time).

3 PFS, or, if induction therapy was reintroduced, addition of the initial PFS and the PFS of the reintroduction.

**Table 2:** Characteristics of other relevant studies included in the pooled-analysis

Source	Scope	Treatment	Sample size	Median age (range) in years	Summary statistics on survival (months)	Criteria for start time for PFS or OS
Tournigand, 2015 (DREAM; OPTIMOX)	Multicenter, France, Austria, and Canada	**Induction:** (BEV + mFOLFOX7/XELOX2/FOLFIRI)/*12 weeks; **Maintenance:** Arm 1: BEV*3 weeks; Arm 2: BEV *3 weeks + Erlo	Arm 1: 228 Arm 2: 224	Arm 1: 63 (57–70) Arm 2: 63 (57–70)	Arm 1: PFS, 4.9 (4.1–5.7); OS, 22.1 (19.6–26.7) Arm 2: PFS, 5.4 (4.1–5.7); OS, 24.9 (21.4–28.9)	PFS: From date of maintenance randomization to first PD. OS: From date of maintenance randomization to death.
Tveit 2012 (NORDIC VII)	Multicenter, Norway, Sweden, Denmark, Iceland	**Induction:** (Cet+ FLOX)/FLOX every 2 weeks *8 cycles; Arm A: FLOX until PD or unacceptable toxicity; Arm B: Cet plus FLOX until PD or unacceptable toxicity; Arm C: Cet; reintroduce FLOX at PD	Arm A: 185 Arm B: 194 Arm C: 187	Arm A: 61.2 (29.9–74.8) Arm B: 60.8 (24.1–74.4) Arm C: 63.6 (33.1–74.9)	Arm A: PFS, 7.9 (7.3–8.5); OS, 20.4 (17.0–23.7) ArmB: PFS, 8.3 (7.4–9.3); OS, 19.7 (15.9–23.5) Arm C: PFS, 7.3 (6.8–7.9); OS, 20.3 (17.3–23.3)	PFS: From random assignment to the first recorded PD or death.
Johnsson 2013 (Nordic ACT)	Multicenter, Denmark, Sweden	**Induction:** (XELOX/XELIRI+BEV) every 3 weeks *6 cycles or (FOLFOX/FOLFIRI+BEV) every 2 weeks *9 cycles; **Maintenance:** Arm1: BEV *3 weeks; Arm2: BEV *3 weeks + Erlo.	Arm 1: 79 Arm 2: 80	Arm 1: 65 (43–82) Arm 2: 64 (48–80)	Arm 1: PFS, 4.23; OS, 22.8 (16.6–25.3) Arm 2: PFS, 5.73; OS, 21.5 (15.4–28.3)	PFS, OS: From start of maintenance treatment.
Waddell 2011 (XelQuali)	Single-arm, Two centers, UK	**Induction:** XELOX every 3 weeks *4 cycles **Maintenance:** Cape *3 weeks	35	58 (38–79)	PFS, 8.1 (6.2–11.8) OS, 23.1 (17.8–28.5)	PFS: From the first day of treatment to first evidence of clinical/radiological PD or death. OS: From registration to death from any cause.
Nakayama 2011 (CCOG-070)	Single-arm, Japan	**Induction:** mFOLFOX6 every 2 weeks *6 cycles **Maintenance:** Oral S-1	21	58 (38–79)	PFS, 7.9; DDC, 9.3; OS, NR	DDC defined as PFS, or, addition of the initial PFS and the PFS of the reintroduction.

Abbreviations: BEV = Bevacizumab; Cape = capecitabine; Erlo = erlotinib; Cet = cetuximab; CFI = chemotherapy-free interval; FLOX = 5-FU/leucovorin/oxaliplatin; FOLFOX = folinic acid (leucovorin)/5-FU/oxaliplatin; XELOX = capecitabine/oxaliplatin; XELIRI = capecitabine/Irinotecan; m = modified; mos = months; NR = not reported; PD = disease progression; 5FU = 5-fluorouracil; PFS = progression-free survival; OS = overall survival; DDC = duration of disease control.

OS was defined as the time from maintenance randomization to death (not including induction therapy time). Data from three trials [8, 10, 11] (*N* = 1,231) were retained for comparison of the maintenance and complete CFI treatment strategies. Of those three trials, data on PFS and OS were available from two [8, 11]; only data on PFS was available from the third [10]. All three trials were superiority trials.

Three trials [9, 12, 13] (*N* = 1,223) were retained for the maintenance vs. continuous treatment groups. Three trials reported PFS, though data on OS were available only from two trials [9, 12] (Note: the 95% confidence intervals [CI] for OS reported by Yalcin et al. were calculated using the imputation method [19]). The three trials were superiority trials.

### Maintenance vs. complete chemotherapy-free interval strategy

**Figure 2:**
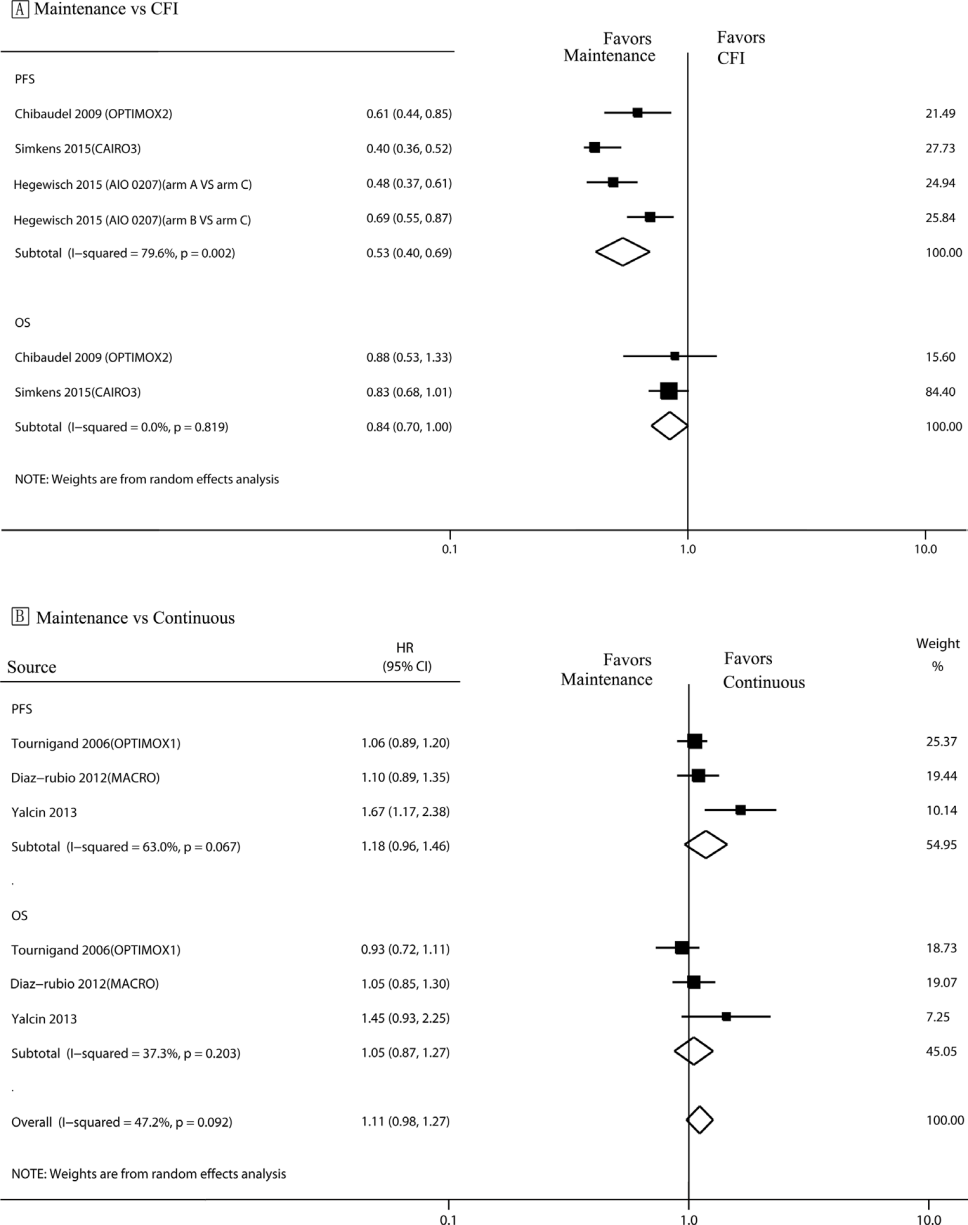
PFS and OS in trials comparing maintenance strategies with CFIs/continuous treatment strategies The size of each data marker correlates with the weighting factor (1/SE^2^) assigned to the study. For the combined results, the length of the diamond represents the 95% confidence interval of the summary. PFS, Progression free survival; OS, overall survival; HR, hazard ratio; CFI, complete chemotherapy-free interval.

Analysis of PFS (Figure 2A) revealed a statistically significant benefit associated with maintenance therapy (hazard ratio [HR]: 0.53, 95% CI, 0.40–0.69) (Figure 2A). No significant difference was observed between the maintenance and complete CFI strategies with respect to OS (HR, 0.84; 95% CI, 0.70–1.00).

### Maintenance vs. continuous strategy

There was no significant inter-group difference between the continuous and maintenance treatment strategies with respect to either PFS or OS (HR: 1.18, 95% CI, 0.96–1.46; HR: 1.05, 95% CI, 0.98–1.27; respectively) (Figure 2B).

### Safety

**Figure 3:**
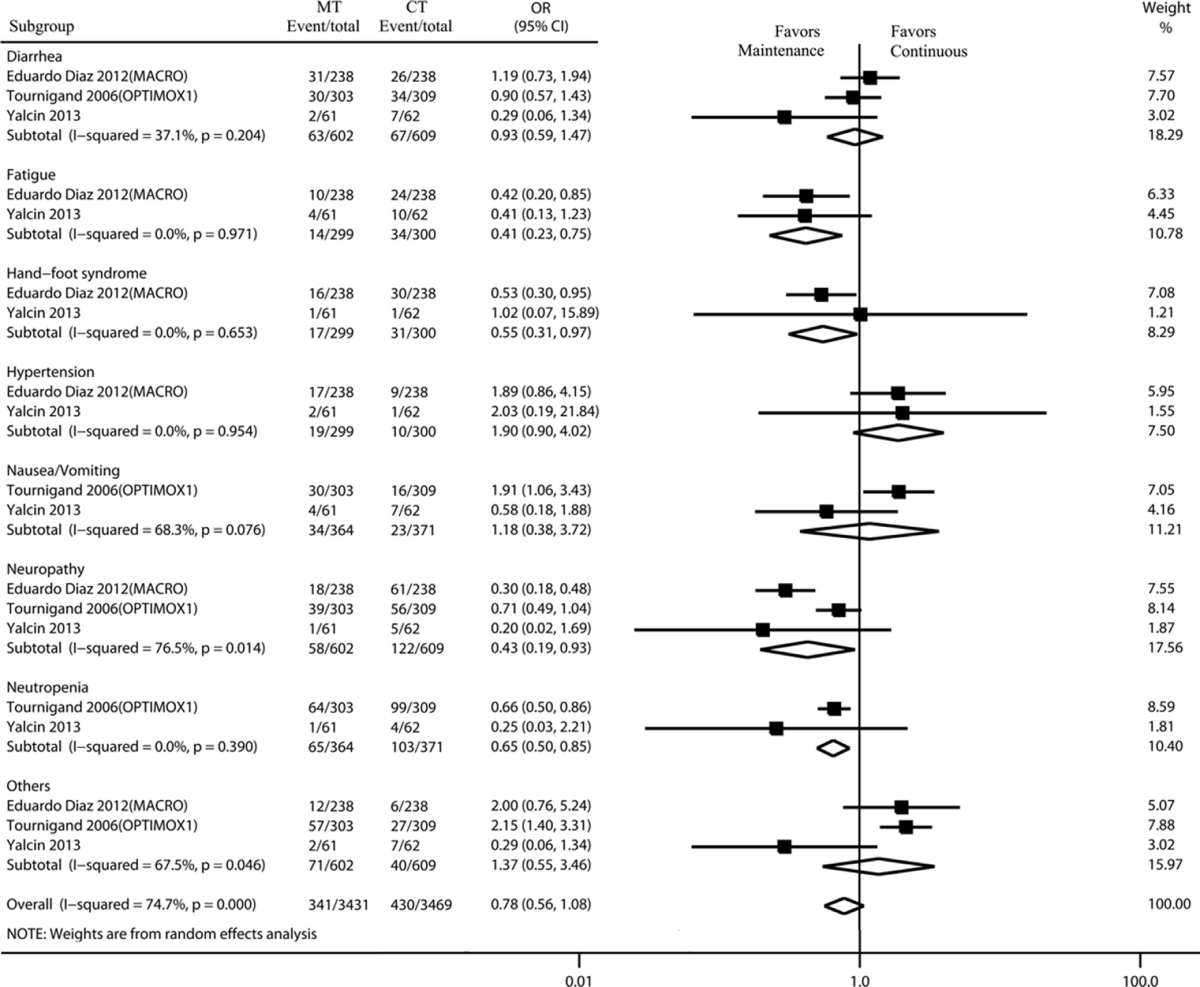
Incidence and relative risk of grade 3 or 4 toxicity with MT and CT Treatment effect was calculated using a random-effect model. OR, Odds ratio; MT, maintenance therapy; CT, continuous therapy.

Adverse events were assessed according to the National Cancer Institute's Common Terminology Criteria for Adverse Events (CTCAE), version 4.0. Grade 3 adverse events included severe or medically significant, but not immediately life-threatening; hospitalization or prolongation of hospitalization indicated; disabling; limiting self-care activities of daily living. Grade 4 adverse events were life-threatening consequences that needed urgent intervention. Four of the five trials reported grade 3 to 4 adverse events. According to CTCAE, severe adverse events reported in ≥ 2 studies or with an incidence > 5% were classified into different system organ classes for analysis. Using a random model, the maintenance strategy was associated with a lower incidence of patients experiencing grade 3/4 adverse events as compared to the incidence associated with the continuous strategy (odds ratio [OR]: 0.78, 95% CI, 0.56–1.08) (Figure 3). The most common grade 3/4 adverse events were neutropenia, neuropathy, and diarrhea. The incidence of neutropenia, neuropathy, hand-foot syndrome and fatigue associated with the maintenance strategy was significantly lower than that associated with the continuous strategy (OR: 0.65, 95% CI, 0.50–0.85; OR: 0.43, 95% CI, 0.19–0.93; OR: 0.55, 95% CI, 0.31–0.97; OR, 0.41, 95% CI, 0.23–0.75; respectively) (Figure 3).

### Heterogeneity and publication bias assessment

No significant heterogeneity was observed in the analyses of OS (*I* ^2^ < 25%, *P* > 0.1). Mild heterogeneity was observed for the comparison of PFS between the maintenance and CFI strategies (25% < *I* ^2^ < 50%, *P* > 0.1). Moderate heterogeneity was observed in the comparison of PFS between the maintenance and continuous strategies (50% < *I* ^2^ < 75%, *P* < 0.1). Neither Begg's test nor Egger's suggested any publication bias that may have influenced the inferences drawn (*P* ranging from 0.17 to 0.99, ranging from 0.11 to 0.86, respectively.

### Pooled analysis of PFS and OS

**Figure 4:**
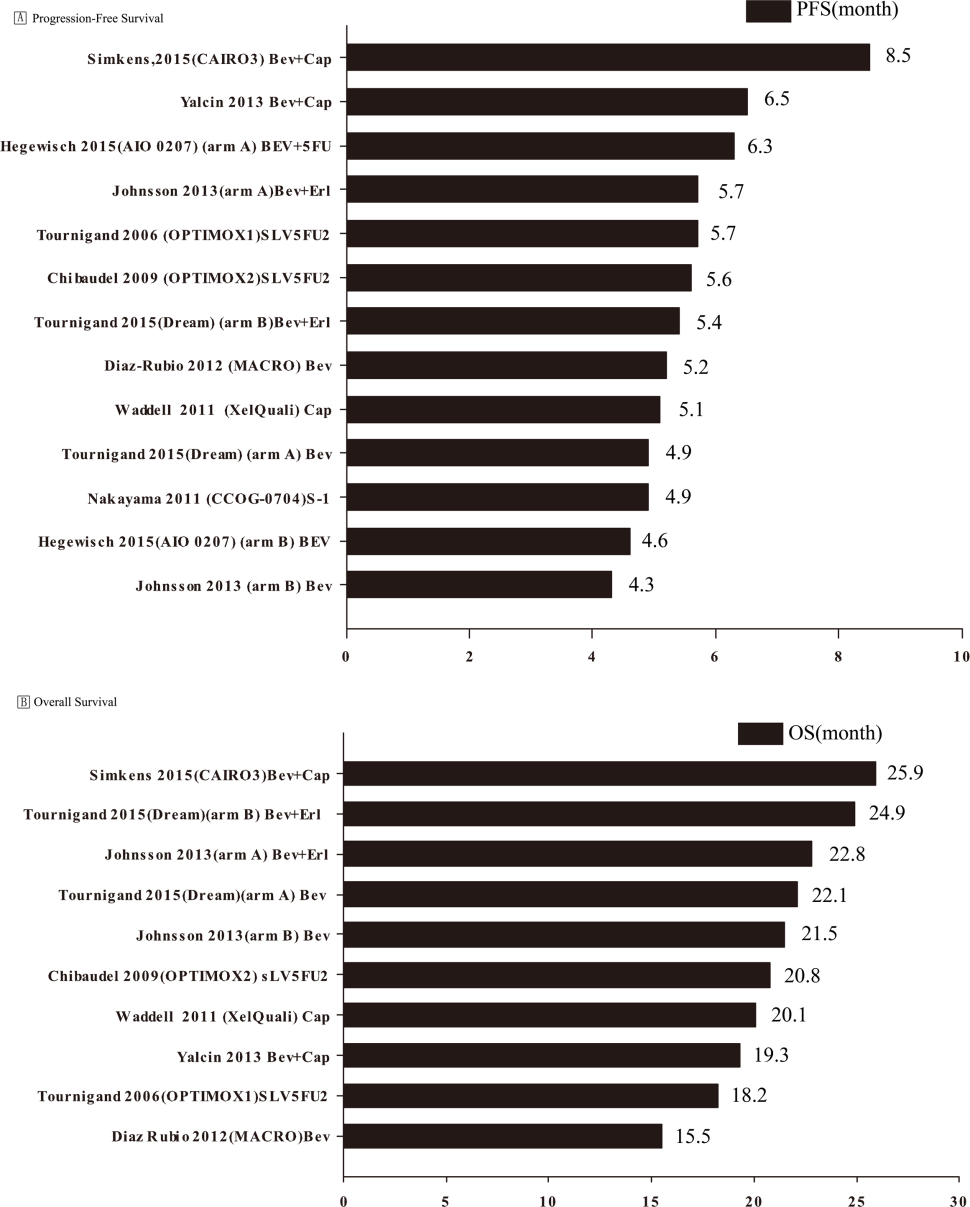
Pooled analysis of progression-free and overall survival BEV, Bevacizumab; Cap, capecitabine; FOLFOX, folinic acid (leucovorin)/5-FU/oxaliplatin; m, modified; Erl, erlotinib; Cet, cetuximab; PFS, progression-free survival; OS, overall survival.

Data on median PFS were available from ten studies [8–16, 18] that evaluated a total of thirteen maintenance therapies (two arms in three trials [11, 14, 16] that were considered as separate trials were all maintenance therapies). Data on median OS were available from eight studies, with a total of ten trials of maintenance therapies (two arms in two trials [14, 16] were maintenance therapies). The mean PFS was 5.59 months (range, 4.30–8.50); the standard deviation, standard error, and variance were 1.07, 0.30 and 1.15, respectively. The mean OS was 21.1 months (range, 15.5–25.9); the standard deviation, standard error, and variance were 3.08, 0.97 and 9.51, respectively (Figure 4).

For PFS, five maintenance regimens [10, 11, 13, 14, 16] were bevacizumab-based doublets (bevacizumab and 5FU/erlotinib/capecitabine), four maintenance regimens [9, 10, 14, 16] employed bevacizumab monotherapy, while four regimens [8, 12, 15, 18] did not employ bevacizumab. The mean PFS associated with bevacizumab-based doublets was 6.48 months as compared to 4.75 and 5.32 months respectively associated with bevacizumab monotherapy and regimens without bevacizumab. One-way ANOVA revealed a significant inter-group difference in PFS between bevacizumab-based doublets and bevacizumab monotherapy (*P* = 0.011), but not significant between other groups.

For OS, four maintenance regimens [11, 13, 14, 16] included bevacizumab-based doublets (bevacizumab and erlotinib/capecitabine), three regimens [9, 14, 16] utilized bevacizumab monotherapy, and three did not use [8, 12, 18] bevacizumab. The mean OS associated with bevacizumab-based doublets was 23.2 months as compared to 19.7 months associated with both bevacizumab monotherapy and regimens without bevacizumab. One-way ANOVA showed no significant inter-group difference with respect to OS.

## DISCUSSION

Compared to the complete CFI treatment strategy, the maintenance strategy had a significant impact on PFS. Moreover, no significant difference was observed between the maintenance and continuous therapies with respect to PFS and OS. However, the incidence of grade 3/4 adverse events tended to be higher with the continuous treatment strategy. In a pooled analysis of data on maintenance treatment, the bevacizumab-based doublets regimen was slightly superior to bevacizumab monotherapy with respect to PFS.

Two systematic reviews have compared the intermittent and continuous chemotherapeutic strategies for mCRC patients [20, 21]. However, the complete CFI and maintenance strategies have basic differences from those approaches. The results of the two OPTIMOX trials [8, 12] indicate that complete CFI is not appropriate, and that non-progressive patients should receive some form of maintenance therapy [22]. The results of our analysis of PFS revealed a statistically significant benefit associated with maintenance over CFI therapy. Furthermore, the data on PFS and the incidence of grade 3/4 adverse events were not factored into the pooled analysis, which precluded assessment of the effect on the patients’ quality of life. Consequently, the scope of those reviews does not allow for comprehensive comparison of maintenance and continuous treatment strategies.

Although drawing definitive inferences and recommendations for an optimal maintenance regimen is difficult, we were able to identify certain key differences with respect to PFS and OS. Much effort has been made to formulate maintenance regimens that prolong the time to progression with minimal toxicity, thereby optimizing the quality of life of mCRC patients. The results of the MACRO trial suggest bevacizumab monotherapy is a valid maintenance option [9]. Yalcin et al. suggested that maintenance therapy with bevacizumab plus capecitabine following induction with 6 cycles of bevacizumab plus XELOX is at least as effective as continuous bevacizumab plus XELOX until progression in patients with previously untreated mCRC [13]. At this year's ESMO meeting, Dirk Arnold presented data from three new clinical trials of maintenance regimens [10, 11, 16]. The newest (AIO 0207) [10] was a de-escalation trial, which showed that maintenance with fluoropyrimidine and bevacizumab provided longer PFS than that associated with de-escalation to bevacizumab monotherapy or to no treatment at all. Any inferences pertaining to OS would be premature at this time. However, continuation of treatment was associated with a longer PFS. One interesting finding was the comparable outcomes attained with bevacizumab monotherapy and a fluoropyrimidine/bevacizumab regimen (median PFS: 4.6 vs. 6.2 months, respectively). To understand this, it is important to note that in the GERCOR DREAM study, the induction treatment (combination chemotherapy plus bevacizumab) was similar in both treatment arms [16], following which de-escalation to bevacizumab monotherapy was compared with an experimental maintenance regimen (erlotinib plus bevacizumab). This was a switch maintenance protocol in which a new drug was integrated. Surprisingly, this approach was associated with a slight improvement in OS. Early pointers from this large randomized trial indicate that erlotinib may be effective for maintenance treatment of patients with mCRC. Another maintenance trial, the Netherlands CAIRO3 trial, revealed a significant improvement in median PFS1 and a slight prolongation of OS with maintenance treatment (bevacizumab plus capecitabine) versus observation [11].

The pooled analysis of PFS demonstrated that maintenance therapy with bevacizumab-based doublets significantly prolonged the time to progression over that associated with bevacizumab monotherapy. There was no significant difference between the two maintenance groups with respect to OS. In the present study, the delay in disease progression did not translate into a statistically significant OS benefit. This may reflect the subsequent strategies as well as the variability in individual characteristics. Following such a strategy, patients unwilling to continue treatment can opt out. However, we believe that most patients are likely to benefit from an active maintenance strategy.

Some notable limitations of our study are worth mentioning. Our meta-analysis was not based on individual patient data. Further, the variability in baseline patient characteristics could not be controlled for. The definition of PFS in some trials was different from the one employed for this meta-analysis. This necessitated adjusting the data according to the study design, which may have been affected by subjective bias.

In conclusion, the efficacy of a maintenance treatment strategy with first-line systemic therapies appears to be comparable to that of continuous treatment. The benefits of a maintenance strategy included reduced incidence of cumulative grade 3/4 toxicity, particularly neutropenia, neuropathy, and hand-foot syndrome. In comparison, maintenance bevacizumab monotherapy appeared to prolong the time to progression.

## MATERIALS AND METHODS

### Data sources and searches

Potentially relevant studies from inception to March 2015 were identified through a structured literature search by two authors independently using Medline, Pubmed (pubmed is a search motor using Medline), EMBASE, Cochrane databases and the meeting abstracts of ASCO and ESMO. The key words employed for the literature search were advanced, metastatic, colorectal cancer, colorectal neoplasms, colorectal tumor, maintenance chemotherapy/strategies/strategy/therapy, stop-and-go, intermittent chemotherapy/strategies/strategy/therapy, continuous chemotherapy/strategies/strategy/therapy and clinical trial. The online abstracts of the retrieved studies were screened for eligibility. The reference lists from the retained articles were manually reviewed to identify any relevant studies missed in the original search. The abstract reports and virtual presentations at the American Society of Clinical Oncology annual meetings and European Society of Medical Oncology congresses from inception to 2014 were searched for relevant studies. Studies were evaluated using the Jadad scoring scale, and articles that scored ≥ 3 points were included in our study.

### Study selection

The eligibility criteria for inclusion of studies were: (1) phase II or III randomized controlled trials involving patients with CRC confirmed by histopathology; (2) studies comparing a maintenance with an intermittent chemotherapeutic strategy or those comparing a maintenance with a continuous chemotherapeutic strategy; (3) one or more of the primary or secondary outcomes were reported. Exclusion criteria were: (1) studies that had only a single treatment arm; (2) data on primary or secondary outcomes not available; or (3) only compared a continuous with an intermittent chemotherapeutic strategy. If more than one study evaluated the same data set, only the most recent publication was included in the meta-analysis. In cases where only the meeting abstract was available, and the article was as yet unpublished, data in the abstracts was supplemented by information from associated materials, including posters and presentation slides.

### Data extraction

Data extraction was performed independently by two authors and entered in a standard data sheet. Data on the following variables were extracted: first author's name, year of publication (acronym of the trial), journal, affiliated institution, country, study phase, format (full text or abstract), interventional and control treatments, hazard ratio (HR) with 95% confidence intervals (CIs) for PFS or OS, median PFS and OS, randomization method, analysis tool, number of patients randomized, demographic and clinical data (age, sex, ethnicity, histology), duration of follow up and toxicity (grade 3/4). Any disagreements were resolved by consensus, if necessary, after involvement of the third author when additional information was required, the corresponding authors were contacted via email.

### Data analysis

All statistical analyses were performed using Stata version 11.2 SE (Stata Corp., College Station, TX, USA) and in compliance to the recommendations of the Cochrane Collaboration). As the outcome proportions were expected to be high, a random effects model was employed to estimate the HR from the pooled data. Meta-analyses were performed for OS and PFS to explore the robustness of the findings across the maintenance and continuous/complete CFI strategies. Two-sided *P* < 0.05 was considered statistically significant. A HR < 1.0 indicated a lower probability of attaining an event in patients receiving maintenance chemotherapy. We used the *X*^2^ test and the *I*^2^ measure for heterogeneity. A probability level for the *X*^2^ statistic < 10% (*P* ≤ 0.10) and/or *I*^2^ > 50% were considered indicative of statistically significant heterogeneity amongst the included trials. In addition, sensitivity analysis was performed to rule out any undue influence of larger studies on the observed associations. This was done by analyzing the results after sequential exclusion of individual larger studies from the meta- analysis.

Finally, we summarized the outcomes of 11 trials through a descriptive statistical analysis. Microsoft Excel was used to generate the graphs (histograms) with horizontal bars for PFS and OS. One-way ANOVA was performed to compare PFS and OS among groups of bevacizumab-based doublets, bevacizumab monotherapy and without bevacizumab.

## SUPPLEMENTARY MATERIALS


