# Supplementary material for: Meta-analysis comparing maintenance strategies with continuous therapy and complete chemotherapy-free interval strategies in the treatment of metastatic colorectal cancer
Source: Oncotarget. 2016 Apr 8;7(22):33418–28. doi: 10.18632/oncotarget.8644 (PMC5078106; doi:10.18632/oncotarget.8644)
Supplement: Supplementary file 1 [file oncotarget-07-33418-s001.pdf]

Meta-analysis comparing maintenance strategies with continuous therapy and complete chemotherapy-free interval strategies in the treatment of metastatic colorectal cancer

Supplementary Materials

Chemotherapy regimens in the trials included in the pool analysis

(1) **XelQuali :**

Induction:XELOX

Oxaliplatin130 mg/m² IV over 2 hoursday 1
Capecitabine 1000 mg/m² twice dailyPOdays 1–14
(total daily dose 2000 mg/m²)
Repeated every 3 weeks

Maintenance:Capecitabine

Capecitabine 1,250 mg/m² twice dailyPOdays 1–14 every 3 weeks until PD

(2) **CCOG-0704:**

Induction:mFOLFOX6

Oxaliplatin85 mg/m²IV over 2 hoursday 1
l-LV 200 mg/m²IV over 2 hours days 1
5-FU 400 mg/m²IV bolus days 1
5-FU2,400 mg/m² IV over 46 hours days 1
Repeated every 2 weeks.

Maintenance: S-1

80 mg for patients with body surface area (BSA) < 1.25 m²;
100 mg for patients with BSA 1.25–1.5 m²
120 mg for patients with BSA ≥ 1.5 m²
(total daily dose was administered orally in two divided dosesfor 28 days, followed by a 14-day treatment-free interval)

(3) **GERCOR DREAM; OPTIMOX3:**

Induction:

BEV+FOLFIRI:

Irinotecan 180 mg/m² 90 minutes infusionday 1
Folinic acid 400 mg/m² racemic form day 1
or 200 mg/m² levo form 2-h infusion
5-FU 400 mg/m² 15-min infusion day 1
5-FU 2400 mg/m² 46-h continuous infusion day 1
BEV: 5 mg/kg IV over 90 minutes day 1
Repeated every 2 weeks

BEV+mFOLFOX7:

Oxaliplatin 100 mg/m² 2-h infusion day 1
Folinic acid 400 mg/m² racemic form or 200 mg/m²levo form 2-h infusion day 1
Fluorouracil 2400 mg/m² 46-h continuous infusion day 1
BEV: 5 mg/kg IV over 90 minutes day 1
Repeated every 2 weeks

BEV+mXELOX:

Oxaliplatin 100 mg/m² 2-h infusion day 1
Capecitabine 1250 mg/m² twice daily PO day 1
(total daily dose 2500 mg/m²)
Repeated every **3** weeks.

Maintenance:

Arm1:BEV 7.5 mg/kg IV over 90 minutes day 1
Repeated every 3 weeks
Arm2:BEV+ Erlo
BEV 7.5 mg/kg IV over 90 minutes day 1
Repeated every 3 weeks
Erlo150 mg oncedaily

(4) **Nordic Act:**

Induction:

BEV+ XELOX

Oxaliplatin130 mg/m² IV over 2 hours day 1
Capecitabine 1000 mg/m² twice daily PO days 1–14
(total daily dose 2000 mg/m²)
BEV 7.5 mg/kg IV over 30 minutes or according to local practice day 1
Repeated every 3 weeks

BEV+ XELIRI:

Irinotecan180 mg/m²90 minutes infusion day 1
Capecitabine 1000 mg/m² bid on daysdays 1–14
(total daily dose 2000 mg/m²)
BEV 7.5 mg/kg IV over 30 minutes or according to local practice day 1
Repeated every 2 weeks

BEV+ FOLFOX:

Oxaliplatin85 mg/m²IV 2 hours day1
Leucovorin200 mg/m²IV 2 hoursday 1
5-FU 400 mg/m²iv bolus day 1
5-FU 2400 mg/m²iv over 44 hours, day1
BEV 5.0 mg/kg IV over 30 minutes or according to local practice day 1
Repeated every 2 weeks

Maintenance:?

(5) CAIRO3

Induction:CAPOX-B:

Capecitabine 1000 mg/m² orally twice daily days 1–14

Oxaliplatin 130 mg/m² intravenously day 1

Bevacizumab 7·5 mg/kg intravenouslyday 1,

Repeated every 3 weeks

Maintenance:Capecitabine

Capecitabine 625 mg/m² orally twice daily days 1–14

Bevacizumab 7.5 mg/kg intravenously days 1

Repeated every 3 weeks

(6) OPTIMOX1

Induction:

FOLFOX4:

Oxaliplatin 85 mg/m²2-hour infusion days1

Leucovorin isomers l-LV 100 mg/m² or dl-LV 200 mg/m² 2-hour infusion day1

5-FU 400 mg/m²iv bolus

5-FU 600 mg/m²iv over 23 hours days 1–2

Repeated every 2 weeks

FOLFOX7:

Oxaliplatin130 mg/m² 2-hour infusion day1

Leucovorin isomers l-LV 200 mg/m² or dl-LV 400 mg/m²2-hour infusion day1

5-FU 2400 mg/m² 46-hour infusion day 1

Repeated every 2 weeks

(s) LV5FU2:

L-LV 200 mg/m² or dl-LV 400 mg/m² 2-hour infusion

5-FU 400 mg/m²iv bolus

5-FU 3,000 mg/m²46-hour infusion

Repeated every2 weeks

(7) AIO 0207

CAPOX:

Capecitabine 1000 mg/m2 twice daily POdays 1–14, 22–35

(total daily dose 2000 mg/m2)

Oxaliplatin 70 mg/m2 IV over 2 hours days 1, 8, 22, 29

Cycle repeated day 43

Bevacizumab 7.5 mg/kg IV over 90 minutes,days 1, 22

if tolerated subsequently over 60and 30 minutes

Repeated day 43

XELOX:

Capecitabine 1000 mg/m2 twice daily POdays 1–14, 22–35

(total daily dose 2000 mg/m2)

Oxaliplatin 130 mg/m2 IV over 2 hours days 1, 22

Cycle repeated day 43

Bevacizumab 7.5 mg/kg IV over 90 days 1, 22 minutes, if tolerated subsequently over 60 and 30 minutes

Repeated day 43

FOLFOX6:

Oxaliplatin 100 mg/m2 IV over 2 hours days 1, 15, 29

(dl-)Folinic acid 400 mg/m2 IV over 2 days 1, 15, 29 hours

5-FU 400 mg/m2 IV bolus days 1, 15, 29

5-FU 2400 mg/m2 IV over 46 hours days 1, 15, 29

Cycle repeated day 43

Bevacizumab 5 mg/kg IV over 90 minutesdays 1, 15, 29

if tolerated subsequently over 60 and 30 minutes

Repeated day 43

FOLFOX4:

Oxaliplatin 85 mg/m2 IV over 2 hours days 1, 15, 29

(dl-)Folinic acid 200 mg/m2 IV over 2 days 1, 2, 15,16, 29, 30 hours

5-FU 400 mg/m2 IV bolus days 1, 2, 15,16, 29, 30

5-FU 600 mg/m2 IV over 22 hours days 1, 2, 15,16, 29, 30

Cycle repeated day 43

Bevacizumab 5 mg/kg IV over 90 minutesdays 1, 15, 29

if tolerated subsequently over 60 and 30 minutes

Repeated day 43

Modified FOLFOX7:

Oxaliplatin 100 mg/m2 IV over 2 hours days 1, 15, 29

(dl-)Folinic acid 400 mg/m2 IV over 2 days 1, 15, 29 hours

5-FU 3000 mg/m2 IV over 46 hours days 1, 15, 29

Cycle repeated day 43

Bevacizumab 5 mg/kg IV over 90 minutesdays 1, 15, 29

if tolerated subsequently over 60 and 30 minutes

Repeated day 43

Modified FOLFOX4:

Oxaliplatin 85 mg/m2 IV over 2 hours days 1, 15, 29

(dl-)Folinic acid 400 mg/m2 IV over 2 days 1, 15, 29 hours

5-FU 3000 mg/m2 IV over 46 hours days 1, 15, 29

Cycle repeated day 43

Bevacizumab 5 mg/kg IV over 90 minutes days 1, 15, 29

if tolerated subsequently over 60 and 30 minutes

Repeated day 43

Simplified FOLFOX4:

Oxaliplatin 85 mg/m2 IV over 2 hours days 1, 15, 29
(dl-)Folinic acid 400 mg/m2 IV over 2 days 1, 15, 29 hours
5-FU 400 mg/m2 IV bolus days 1, 15, 29
5-FU 2400 mg/m2 IV over 46 hours days 1, 15, 29
Cycle repeated day 43
Bevacizumab 5 mg/kg IV over 90 minutes, days 1, 15, 29
if tolerated subsequently over 60 and 30 minutes
Repeated day 43

(8) OPTIMOX2

Modified FOLFOX7:

Oxaliplatin100 mg/m^2 2-hour infusion day1
LV 400 mg/m^2 2-hour infusion day1
5-FU 3000 mg/m^2 48-hour infusion day 1
Repeated every 2 weeks

Simplified LV5FU2:

LV 400 mg/$m^2$2-hour infusion day1
5-FU 400 mg/m^2 IV bolus day1
5-FU 3000 mg/m2 48-hour infusion day1
Repeated every 2 weeks

(9) MACRO

Induction:

XELOX:

Oxaliplatin 130 mg/m^2 day 1
Capecitabine 1,000 mg/m^2 twice daily days 1–14
Bevacizumab 7.5 mg/kg day 1
Repeated every 3 weeks

(10) Yalcin 2013

Induction:

XELOX:

Oxaliplatin 130 mg/m^2 IV on day 1
Capecitabine 1,000 mg/m^2 orally twice daily days 1–14
bevacizumab 7.5 mg/kg day 1
Repeated every 3 weeks

(11) NORDIC VII

Arm A: FLOX

Regimen 23 was administered every 2 weeks as
Oxaliplatin 85 mg/m^2 over 1 hour (30 to 90 minutes) day 1
5-FU 500 mg/m^2 IV bolus (<5 minutes) day 1 and 2
FA 60 mg/m^2 IV bolus (<10 minutes) days 1 and 2
Repeated every 2 weeks

Arm C: FLOX+Cetuximab

Cetuximab was administered every week as an initial dose of 400 mg/m^2 and there after 250 mg/m^2.
